# Switching from Intravenous to Subcutaneous Infliximab in Psoriasis: A Case Series on Efficacy and Treatment Satisfaction

**DOI:** 10.3390/jcm14248875

**Published:** 2025-12-15

**Authors:** Daniele Rizzo, Gaetano Licata, Massimo Frazzitta, Leonardo Zichichi

**Affiliations:** U.O.C. Dermatologia, Ospedale S. Antonio Abate, ASP Trapani, Via Cosenza, 82—Casa Santa Erice, 91100 Trapani, Italy; gaetano.licata89@gmail.com (G.L.); mfrazzitta@virgilio.it (M.F.); l.zichichi@gmail.com (L.Z.)

**Keywords:** plaque psoriasis, monoclonal antibody, anti TNF-α, infliximab, Psoriasis Area and Severity Index (PASI), Dermatology Life Quality Index (DLQI) and Physician Global Assessment (PGA), Treatment Satisfaction Questionnaire Medication-9 (TSQM-9)

## Abstract

**Background/Objectives:** Plaque psoriasis is a chronic immune-mediated inflammatory disease affecting approximately 3% of the global population and resulting in a significant deterioration in quality of life. Systemic therapy with monoclonal antibodies (mAbs) targeting TNF-α, IL-23, and IL-17 improves clinical outcomes and patients’ quality of life. Treatment strategies commonly include different mAbs and different sequencing approaches between agents, which are well-established in clinical practice. In contrast, evidence supporting the switch from intravenous to subcutaneous administration of the same mAb remains limited. Herein, we report data from a retrospective case series of patients with plaque psoriasis treated with intravenous infliximab (IV-IFX; Anti TNF-α) and transitioned to subcutaneous infliximab (SC-IFX) to compare clinical and patient-reported outcomes across routes. **Methods:** A total of 11 plaque psoriasis patients were retrospectively analyzed. The scores of the Psoriasis Area and Severity Index (PASI), Dermatology Life Quality Index (DLQI), and Physician Global Assessment (PGA) were assessed during IV-IFX and after switching to SC-IFX. To evaluate patients’ satisfaction, the Score of Treatment Satisfaction Questionnaire Medication-9 (TSQM-9) was evaluated. Both Student’s *t*-test and ANOVA were used to assess statistically significant differences between the two routes of administration (*p* < 0.05). **Results:** Scores for PASI, DLQI, and PGA were lower with SC-IFX compared with IV-IFX, indicating improved disease control and quality of life after the switch. PASI and DLQI improved in 81% and 100% of patients treated with SC-IFX, respectively. TSQM-9 total scores increased significantly by 24% (*p* < 0.001). In particular, the questions addressing the “convenience” of treatment revealed a marked advantage for the SC-IFX formulation (*p* < 0.001). No treatment-emergent adverse events were registered. **Conclusions:** In this retrospective case series, switching from IV-IFX to SC-IFX appeared to be safe and to maintain or improve clinical response and enhanced treatment satisfaction. These findings highlight the potential of SC-IFX as a viable maintenance option for patients with plaque psoriasis previously treated with IV-IFX.

## 1. Introduction

Psoriasis is a chronic disease caused by an inflammatory condition of the skin, sometimes associated with other conditions such as psoriatic arthritis, psychological, cardiovascular, and gastrointestinal disorders. The World Health Organization (WHO) [1] has considered psoriasis a serious non-communicable disease and has emphasized the importance of appropriate clinical treatments, which are often compromised due to frequent misdiagnosis [2]. Psoriasis occurs in both sexes but tends to manifest earlier in women and in those with a family history of the disease. The age of onset usually falls within two decades: between 30 and 39 years and between 60 and 69 years for men, whereas in women, the first symptoms may appear up to 10 years earlier [3,4]. The clinical manifestations of psoriasis are heterogeneous and include cutaneous and systemic manifestations [5,6]. Most commonly, psoriasis presents as plaque psoriasis (psoriasis vulgaris), occurring in 80% of cases [5,6]. Symptoms include itching, burning sensations, and skin tenderness [6,7]. Several comorbidities are associated with psoriasis, including psoriatic arthritis, cardiovascular disease, metabolic syndrome, obesity, and inflammatory bowel disease [6,7,8]. Due to these systemic implications, psoriasis is also referred to as a systemic disease [4], a pathology linked to skin barrier dysfunction [9] and metabolic syndrome [10].

The etiology of psoriasis is complex and encompasses many intrinsic and extrinsic factors [6,11,12,13,14]. Psoriasis is mainly characterized by the activation of inflammatory pathways in innate and adaptive immune cells, leading to uncontrolled keratinocyte (KC) proliferation, acanthosis, neovascularization, and dense infiltration of immune cells into the skin [15]. Pro-inflammatory cytokines primarily involved in the pathogenesis of psoriasis are mainly produced by T cells (e.g., IL-17, IL-21, IL-22, IFN-γ) and dendritic cells (DCs; e.g., TNF-α, IL-6, IL-20, IL-23, NO), forming a central pathogenic loop with IL-23-producing dendritic cells, IL-17-producing Th17 cells, and activated keratinocytes (KCs) [4,5,6,11]. TNF-α initiates the inflammatory cascade in psoriasis. It is secreted in response to triggers such as skin lesions, environmental stimuli, auto-antigens, and TLR receptor agonists [16,17], mainly by activated T cells and antigen-presenting cells (APCs) [18,19]. TNF-α synergizes with IFN-γ to induce chemokine expression in endothelial cells, promoting immune cell infiltration [6,20] and stimulating KCs and DCs to secrete IL-23 [21]. Accordingly, the results of numerous genome-wide association studies (GWAS) and clinical trials support the central role of the inflammatory signaling molecular pathways of TNF-α, IL-23, and IL-17 as the “founding fathers” of the pathogenesis of psoriasis, particularly plaque psoriasis [14,22,23,24].

Both the Food and Drug Administration (FDA) and the European Medicine Agency (EMA) have approved different monoclonal antibodies (mAbs) for treating plaque psoriasis, targeting TNF-α (e.g., adalimumab, etanercept, infliximab) [25,26], IL-23 (e.g., risankizumab, tildrakizumab, guselkumab) [27,28], and IL-17 (e.g., secukinumab, brodalumab, bimekizumab, ixekinumab) [14,23,29]. These agents achieve PASI 90 and PASI 100 response rates between 67% and 89% [14].

Because of this extensive range of options, several international consensus guidelines support a personalized approach to care [30,31,32,33,34,35,36], accounting for disease phenotype, treatment history, comorbidities (e.g., IBD, demyelinating disease, drug characteristics (e.g., administration route, frequency), and lifestyle factors (e.g., family planning) [30,37,38,39,40,41,42]. For instance, genomic data, particularly the HLA*C60:02 status, may also guide treatment selection [43]; HLA*C60:02-negative patients tend to respond better to anti-TNF-α than anti-IL12/23 agents [43].

Furthermore, the availability of administration routes that do not require the assistance of specialized healthcare personnel, such as subcutaneous formulations or oral options, may offer a practical advantage for simplifying clinical management and improving patient convenience [44].

Real-world evidence (RWE) from immune-mediated diseases, including inflammatory bowel diseases (IBDs), Spondyloarthritis (SpA), Rheumatoid Arthritis (RA), psoriatic arthritis (PsA), and chronic plaque psoriasis (PsO), supports the clinical utility of SC infliximab, showing high treatment persistence, stable disease control, and favorable safety profiles in routine practice, together with improvements in convenience and patient experience [45,46,47,48,49,50,51,52]. Notably, these effects are likely attributable to the stable pharmacokinetic profile of the subcutaneous formulation, characterized by high Ctrough values and reduced immunogenicity [53]. These data reinforce the rationale for evaluating an IV-to-SC transition in plaque psoriasis, where comparable benefits, particularly around convenience and adherence, may be clinically meaningful.

Building on this rationale, the present study reports real-world outcomes on switching to and maintaining SC infliximab monotherapy in patients with plaque psoriasis.

## 2. Materials and Methods

### 2.1. Study Population

Data were collected between December 2023 and January 2024 and entered in an ad hoc database containing anonymized clinical and personal data. Anonymization was ensured through the immediate generation of an encrypted code, guaranteeing patient confidentiality.

### 2.2. Clinical Condition, Comorbidities, and Data Recovery

Data from 11 patients were entered into our database for statistical analysis. All patients had moderate to severe plaque psoriasis vulgaris. The patient group for these analyses consisted of 6 females and 5 males. The mean age was 45.5 ± 18.2 years. The diagnosis of plaque psoriasis was made at a specialized dermatology center. All patients were referred for systemic treatment, and each patient received intravenous IFX prior to transitioning to subcutaneous IFX administration. No other treatments, either concurrent or alternative, were administered. Only one patient (ID 4) in this case series presented with comorbid psoriatic arthritis.

### 2.3. Treatment

All patients had previously failed one or more courses of traditional systemic therapies (e.g., retinoids, cyclosporine, methotrexate) and subsequently began therapy with intravenous IFX at a dose of 5 mg/kg, with a mean treatment duration of 20 months (range: 9–38 months). According to the product information, transition to SC IFX therapy can begin from the sixth week of therapy, following two intravenous IFX infusions—one at initiation and one after two weeks. In this case series, all patients received intravenous IFX administered by trained personnel. Thereafter, SC-IFX was self-administered by the patients themselves, as reported in the literature [54]. All patients transitioned to SC-IFX after a mean of 20–27 months of intravenous IFX and continued SC therapy for 12 months.

### 2.4. TSQM-9 Questionnaire

Patient satisfaction with their pharmacological treatment was assessed using the Treatment Satisfaction Questionnaire Medication-9 (TSQM-9). The questionnaire consists of 9 questions covering various aspects of treatment and offers single-choice, multiple-option answers on Likert scales (only one answer is selectable). Seven of the nine questions use a 7-point Likert scale, ranging from 1 (bad) to 7 (good), while two questions (Q7 and Q8) use a 5-point Likert scale, ranging from 1 (bad) to 5 (good). Lower scores correspond to worse impressions, while higher scores are associated with better impressions. The total score of the questionnaire ranges from 9 (minimum) to 59 (maximum) points. A total score of 50 points indicates a high level of patient satisfaction (HLS). However, based on procedures reported in the scientific literature, TSQM-9 results are expressed as percentages (0–100%) to allow for data normalization [55]. Patient-reported scores were normalized by expressing the maximum questionnaire score (59 points) as 100%. Indeed, TSQM-9 values ≥50 points (84.75%) were selected as the threshold for HLS. TSQM-9 data were collected at the end of IV-IFX treatment and after 12 months of SC-IFX treatment.

### 2.5. PASI, DLQI, and PGAs

The Psoriasis Area and Severity Index (PASI), Dermatology Life Quality Index (DLQI), and Physician Global Assessment (PGA) scores were assessed before and after SC-IFX treatment. The PASI (0–72), DLQI (0–30) and the PGA (0, clear; 1, almost clear; 2, mild; 3, moderate; and 4, severe; Table 1) scores recorded by the patient were assessed by the attending physician at SC-IFX treatment initiation and 48 weeks after SC-IFX treatment [56].

### 2.6. Statistical Analyses

Statistical analyses were performed using GraphPad 8.0 version (PRISM, San Diego, CA, USA). The Shapiro–Wilk test was applied to determine whether the data were parametrically distributed. Both W- and *p*-values were calculated. Student’s t-Test (parametric and paired) was used to compare TSQM-9 after IV-IFX (0 weeks) and SC-IFX (48 weeks) visits. Student’s t-Test was used to compare TSQM-9 scores, their normalized values (expressed as percentages), and delta variations between both treatments relative to the HLS reference point. In addition, ANOVA was used to compare TSQM scores between IV and SC–IFX treatments. All results are expressed as the mean ± standard deviation (SD) and number of items (N). For all quantitative analyses, statistical significance was defined as a *p*-value less than 0.05 (*p* < 0.05, *: *p* < 0.01, **; *p* < 0.001, ***; and *p* < 0.0001, ****).

## 3. Results

### 3.1. Follow-Up and Treatments

**Figure 1:**
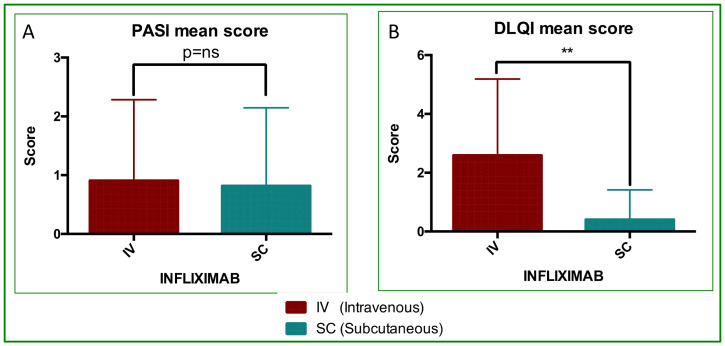
PASI and DLQI analyses. Mean score value and statistical analyses performed by paired Student’s and Wilcoxon tests. (**A**) PASI, differences were not significantly different; *p* = ns. (**B**) DLQI, differences were significantly different (*p* = 0.0041, **).

All patients demonstrated full treatment compliance with SC-IFX self-injections, and all completed at least 12 months of follow-up. The mean total treatment time (TTT; IV + SC) was 32.4 months (22 ÷ 50 months). Both PASI (Figure 1A) and DLQI (Figure 1B) improved at follow-up: the mean PASI decreased by approximately 10% after SC treatment, and DLQI improved in 100% of patients. PASI was better in 3/11 (27.3%), stable in 6/11 (54.5%), and worse in 2/11 (18.2%). Therefore, a total of 81.8% of patients did not show any worsening in their PASI scores. Notably, the two patients with worsening had low absolute PASI scores (2 and 4, respectively; Table 1). No adverse events were reported (Table 2).

### 3.2. TSQM Analyses

**Figure 2:**
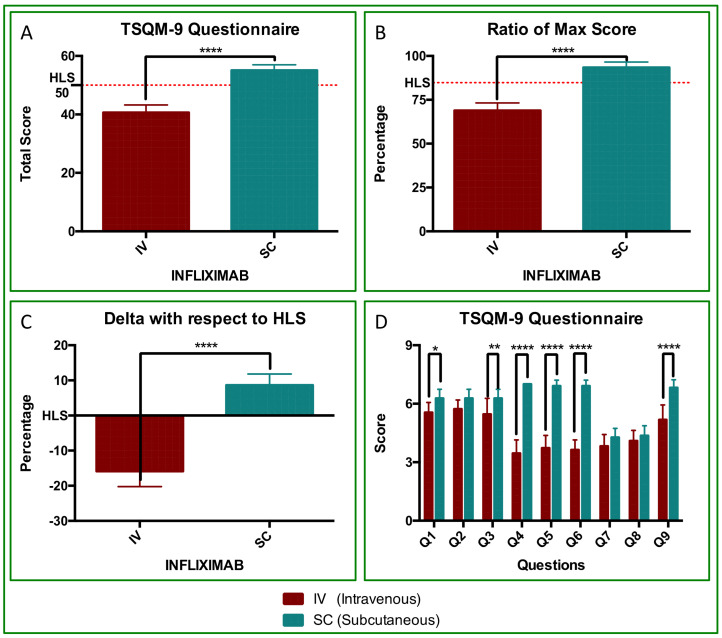
Treatment satisfaction (TSQM-9) with IV-IFX vs. SC-IFX. (**A**) TSQM-9 total score (0–59). The dotted line marks the HLS threshold (50/59 points). (**B**) Normalized TSQM-9 values (%), with a maximum of 100% (59 points). The dotted line marks the HLS threshold (84.75%). Differences were statistically significant (*p* < 0.0001, ****). All SC patients scored above 50 points. (**C**) Delta variation from the HLS threshold, showing negative values for IV-IFX and positive values for SC-IFX (*p* < 0.0001). (**D**) TSQM-9 Questionnaire (Q1–Q9 comparison). ANOVA with Sidak’s post hoc test showed statistically significant treatment effects and differences in mean item scores (*p* < 0.0001). Differences were highly significant for Q1 (*p* < 0.05, *), Q3 (*p* < 0.01, **), Q4 to Q6 (*p* < 0.0001,****), and Q9 (*p* < 0.0001, ****).

The TSQM-9 revealed a highly significant difference between IV-IFX and SC-IFX (Figure 2). Mean TSQM scores were 40.6 ± 0.8 for IV-IFX and 55.1 ± 0.6 for SC-IFX (*p* < 0.0001; Figure 2A). Scores were normalized to 0–100% to align with the existing literature, as detailed in the Section 2. The resulting percent TSQM values were 68.9 ± 4.4 in IV and 93.4 ± 3.2 in SC, respectively (*p* < 0.0001; Figure 1B). With respect to the HLS threshold, the deltas were −15.9% ± 4.4 for IV-IFX and 8.6% ± 3.2 for SC-IFX with a mean between-treatment difference of 24.5% (*p* < 0.0001; Figure 2C). Considering all items (Q1-Q9), the ANOVA results indicate significant effects of treatment (IV vs. SC; *p* < 0.0001) and differences in item scores (*p* < 0.0001). Sidak post hoc testing identified significant differences in 6 of 9 items: Q1 (*p* < 0.05), Q3 (*p* < 0.01), Q4 (*p* < 0.0001), Q5 (*p* < 0.0001), Q6 (*p* < 0.0001), and Q9 (*p* < 0.0001). Notably, Q4 to Q6, which assess treatment management/convenience, favored SC-IFX treatment, with mean differences of 3.545, 3.182, and 3.273 for Q4, Q5, and Q6, respectively (*p* < 0.0001; Figure 2D).

### 3.3. PGA

**Figure 3:**
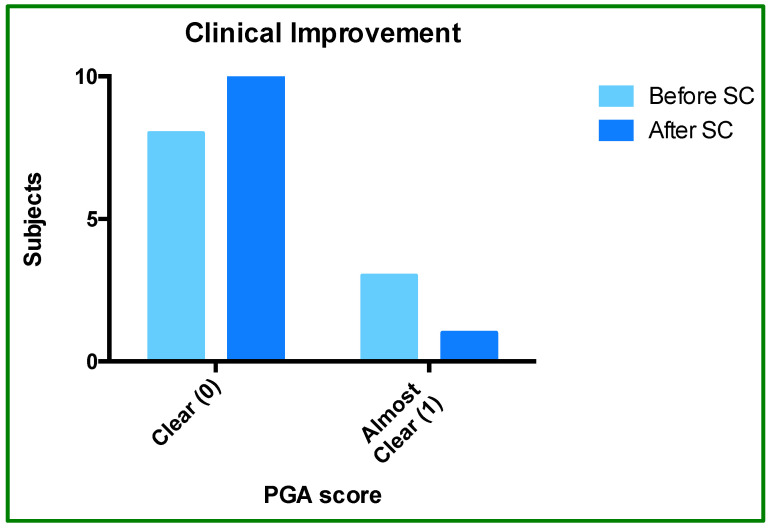
Physician Global Assessment (PGA) before and after SC-IFX initiation. The bar graph shows the proportion of patients rated as “clear” (score of 0) or “almost clear” (score of 1) before and after switching to SC-IFX.

PGA score analysis showed an improvement, indicating an added benefit of SC-IFX. The proportion of patients who rated clear (score 0) versus almost clear (score 1) was 8:3 during IV treatment and 10:1 after SC treatment. This pattern reflects an interesting trend: SC-IFX did not worsen treatment clinical status and was rather associated with improvement in 18.2% of subjects (Figure 2 and Figure 3).

### 3.4. Adverse Events (AEs)

Treatment-emergent adverse events (AEs), defined as any untoward medical occurrence from the first administration of SC IFX to the end of follow-up, were recorded. In line with safety data for intravenous infliximab, particular attention was paid to infusion/injection-site reactions, infections, hypersensitivity reactions, and other treatment-related systemic events. In this case series, no adverse reactions were observed in any patient after switching.

## 4. Discussion

Inhibitors targeting TNF-α, IL-17, and IL-23 are widely used in clinical practice for the maintenance treatment of moderate-to-severe psoriasis [4,14,20,29,54,56]. The present study demonstrated the efficacy and safety of subcutaneous infliximab (SC-IFX) following long-term intravenous infliximab (IV-IFX) treatment. Over a mean follow-up of 32.4 months (Table 1), SC-IFX was well tolerated and associated with maintained or improved clinical and patient-reported outcomes compared with prior IV-IFX exposure.

After switching, the mean PASI and DLQI values were 0.82 and 0.00, respectively (Table 1 and Figure 1). This is consistent with the literature thresholds in which successful treatment corresponds to near-zero PASI and DLQI scores [51]. The change in PASI after the switch was not statistically significant, which was expected given the already very low switch PASI values. This indicates that SC treatment effectively maintained clinical remission. In contrast, DLQI showed a significant additional improvement (*p* = 0.0041; Figure 1), reflecting further gains in patient-reported quality of life after the switch.

To contextualize our TSQM-9 findings, we compared our satisfaction scores with those reported in another real-world psoriasis cohort using the same questionnaire, rather than to directly compare different therapies. In our study, patient satisfaction assessed with the TSQM-9 questionnaire was high, with an overall satisfaction score of 93%. For reference, the ProLOGUE study—an open-label, multicenter, real-world randomized study in Japanese adults with plaque psoriasis treated with the IL-17RA inhibitor brodalumab via subcutaneous injection—reported a TSQM-9 satisfaction score of 83% [55].

Furthermore, in our study, the TSQM-9 results also surpassed the high-level satisfaction (HLS) threshold, both in absolute score (50/59 points) and percentage (84.74%). ANOVA analysis showed that items Q4, Q5, and Q6, which reflect “treatment management/convenience” from the patient’s point of view [56], were 3-points higher on average with SC-IFX than those registered for IV-IFX (*p* < 0.001; Figure 2D). This improvement, although numerically modest, represents a statistically and clinically meaningful enhancement in patients’ day-to-day treatment experience and exceeded the outcomes reported in the ProLOGUE trial [55].

Finally, PGA analyses showed a trend favoring SC-IFX, suggesting that route switching did not hinder the physician-assessed outcomes (Figure 2). These findings are consistent with real-world evidence (RWE) from other chronic immune-mediated diseases, such as inflammatory bowel diseases (IBDs, psoriatic arthritis (PsA), Spondyloarthritis (SpA), Rheumatoid Arthritis (RA), and chronic plaque psoriasis (PsO) [48,49,50,51,52,54,55,56]. In these instances, SC-IFX has been associated with high long-term treatment persistence, improved patient convenience and quality of life, lower immunogenicity, and improved cost-effectiveness. Although we did not perform pharmacokinetic or immunogenicity assessments in our cohort, phase I–III bridging studies comparing SC-IFX with its intravenous formulation have shown higher and more stable trough serum concentrations with SC administration, while maintaining comparable efficacy, safety, and overall rates of anti-drug antibodies [58,59,60]. This pattern has been interpreted as compatible with a high-zone tolerance mechanism, whereby sustained higher drug exposure does not increase, and may attenuate, clinically relevant immunogenic responses [53]. This pharmacokinetic and immunogenicity profile provides a mechanistic rationale for our observation that switching from IV to SC infliximab maintained clinical remission in patients with psoriasis.

Intriguingly, the recent REWATCH study provides reassuring data that earlier switching from IV- to SC-IFX does not compromise efficacy and safety in IBD patients [46]. Further evidence is needed to explore this hypothesis in the context of other chronic immune-mediated diseases. This case series provides proof of principles for the feasibility and safety of switching psoriasis patients from IV-IFX to SC-IFX. However, given the limited sample size and retrospective design, these results should be interpreted with caution, and larger comparative studies will be needed to confirm our findings. Despite these limitations, the data show high long-term treatment persistence, aligning with other real-world evidence across chronic immune-mediated diseases [46], reinforcing the potential of SC-IFX formulation to increase patient satisfaction and improve overall disease management.

## 5. Conclusions

In conclusion, switching from intravenous to subcutaneous infliximab treatment was associated with reductions in DLQI and PGA scores, while maintaining similar PASI scores, in patients with plaque psoriasis. TSQM-9 scores revealed a greater treatment satisfaction with SC-IFX, particularly regarding convenience and daily treatment management. The results suggest that switching to subcutaneous IFX therapy did not worsen clinical assessment scores or raise safety concerns, supporting SC-IFX as a valid and apparently safe maintenance option after prior exposure to IV-IFX. Despite the modest sample size (*n* = 11) and the need for further comparative studies to validate these findings, these data underscore the potential of subcutaneous IFX to optimize long-term disease management in patients with plaque psoriasis previously treated with intravenous IFX.

## Figures and Tables

**Table 1:** Physician Global Assessment (PGA) [57].

Score	Category	Description
0	Clear	No sign of plaque psoriasis
1	Almost clear	Just perceptible erythema and just perceptible scaling
2	Mild	Light pink erythema with minimal scaling with or without pustules
3	Moderate	Dull red, clearly distinguishable erythema with diffusing scaling, some thickening of the skin, with or without fissures, with or without pustule formation
4	Severe	Deep, dark red erythema with obvious and diffuse scaling and thickening, as well as numerous fissures with or without pustule formation

**Table 2:** PASI and DLQI scores reported for IV-IFX and SC-IFX treatment.

PT ID	Demographics		Diagnosis	Infliximab IV Treatment			Infliximab SC Treatment			TTT
	Age	Gender		Months	PASI	DLQI	Months	PASI	DLQI	
1	23	F	Plaque Psoriasis	38	4	4	12	0	0	50
2	54	F	Plaque Psoriasis	16	2	2	12	0	0	28
3	47	M	Plaque Psoriasis	9	0	2	12	0	0	22
4	49	F	Plaque Psoriasis	12	2	4	12	1	0	24
5	66	M	Plaque Psoriasis	11	2	4	12	2	0	23
6	52	M	Plaque Psoriasis	28	0	0	12	0	0	40
7	22	F	Plaque Psoriasis	19	0	4	12	0	0	31
8	42	M	Plaque Psoriasis	30	0	6	12	4	0	42
9	32	F	Plaque Psoriasis	16	0	5	12	0	0	28
10	81	M	Plaque Psoriasis	22	0	8	12	2	0	34
11	32	F	Plaque Psoriasis	22	0	8	12	0	0	34
Mean	45.5			20.27	0.91	4.27	12	0.82	0.00	32.36

Note: IV, intravenous; SC, subcutaneous; PASI, Psoriasis Area Severity Index; DLQI, Dermatology Life Quality Index; TTT, Total Treatment Time.

## Data Availability

Data are available from the corresponding author upon request.
